# Evaluating the efficacy of tuberculosis Advocacy, Communication and Social Mobilization (ACSM) activities in Pakistan: a cross-sectional study

**DOI:** 10.1186/1471-2458-13-887

**Published:** 2013-09-25

**Authors:** Tahir Turk, Fiona J Newton, Joshua D Netwon, Farah Naureen, Jodah Bokhari

**Affiliations:** 1 Communication Partners International, 24 Dulwich Road, Springfield 2250, NSW, Australia; 2 Curtin University School of Public Health, Curtin University, GPO Box U1987, Perth 6845, WA, Australia; 3 Department of Marketing, Peninsula Campus, Monash University, Frankston, VIC 3199, Australia; 4 MercyCorps (Pakistan), 36, Street 1, Islamabad F-6/3, Pakistan; 5 Communications Consultant, Islamabad, Pakistan

**Keywords:** Tuberculosis, Campaign evaluation, Pakistan, Health communication, Advocacy, Communication and Social Mobilization (ACSM) activities

## Abstract

**Background:**

Tuberculosis (TB) continues to be a major public health and development problem within many low- and middle-income countries. Although Advocacy, Communication and Social Mobilization (ACSM) activities have been undertaken in high TB burden countries to remediate these issues, there is little empirical evidence of the efficacy of these approaches. The purpose of this study was therefore to examine the efficacy of an ACSM program undertaken within Pakistan. Pakistan was chosen because it has received considerable funding for ACSM related activities and is one of 22 high-burden TB countries.

**Methods:**

The program was evaluated by surveying a stratified random sample of 2,400 participants across 57 districts of Pakistan. Participants were categorized into one of three groups: aware of both media and community ACSM activities (Aware_Media_&_Community_), aware of ACSM media activities only (Aware_Media_), or unaware of any ACSM activities (Unaware_Media_&_Community_).

**Results:**

Independent measures ANCOVA revealed complex differences in knowledge, attitudes, and intended behaviors towards TB between the three groups. In general, Unaware_Media_&_Community_ cases had a poorer understanding of TB and its treatment, whilst awareness of ACSM activities was highest among literate and urban dwelling Pakistanis. Preferred sources of TB information were also found to vary by gender, geographic location, and literacy.

**Conclusions:**

Whilst highlighting improvements in knowledge and attitudes toward TB, the results also provide invaluable insights into areas where further work needs to be done to address deficits in TB understanding, particularly among rural and illiterate Pakistanis. Equally important, the findings have implications for future TB ACSM initiatives in Pakistan in terms of leveraging the preferred media channels of key demographic segments and exploring the degree to which exposure to multiple channels of communication may have an additive effect on health knowledge.

## Background

Tuberculosis (TB) remains a major public health issue in many developing nations [1]. Lack of public awareness, coupled with limited engagement of communities and non-governmental organizations, have been identified as challenges impeding TB control [2]. To address these challenges, the World Health Organization recommends the utilization of an Advocacy, Communication and Social Mobilization (ACSM) framework for national TB programs [3]. This strategy framework addresses four key challenges: improving case detection and treatment adherence, reducing stigma and discrimination, empowering TB patients, and mobilizing the resources and political commitment required to combat TB [3]. As such, ACSM activities are an important and necessary step in achieving universal access to high quality care for those suffering with TB [4].

Notwithstanding the exigency of ASCM program initiatives, little empirical data has been obtained regarding how communities interact with ACSM messages and program activities. This study therefore seeks to explore the community impact of ACSM activities in Pakistan. Pakistan was selected as the context for this study because it is among 22 designated high burden TB countries, ranking fourth in terms of TB mortality rates (34 deaths per 100,000 population) and fifth in terms of incidence (231 cases per 100,000 population) [1]. Additionally, the country is still struggling to meet targets from the World Health Organization for 70% case detection and 85% cure rates in many areas [5].

In 2007, a 6-year ACSM TB program was initiated in Pakistan by MercyCorps (Pakistan) with funding from the Global Fund to Fight AIDS, Tuberculosis and Malaria. The program was rolled out to 57 districts across the four provinces of Pakistan and the territory of Azad Jammu and Kashmir, an area encompassing approximately 70.5 million people [6]. The first 3 years of the program have been directed towards: (i) conducting mass media and community-based TB communication campaigns; (ii) educating journalists, policy makers, opinion leaders, and service providers about TB; (iii) building coalitions between non-governmental organizations and community-based organizations; and (iv) producing and disseminating resource materials. This study will focus on evaluating the public-facing ACSM activities (i.e., the mass media and community-based communication campaigns).

The mass media and community-based 'Stop TB’ campaigns were initiated to improve knowledge about the diagnosis, treatment, and prevention of TB, and to increase awareness about the widespread availability of directly observed treatment, short-course (DOTS) services for TB [6]. The objective was to empower communities and people with TB to actively engage with diagnostic and treatment programs. The campaigns were targeted toward males and females aged 18–49 years residing in urban and rural areas.

The primary objective of this study is to determine whether the TB knowledge and attitudes of target audiences differ as a function of their recall of the ACSM media and community communication-based campaigns. This approach corresponds to evaluations of lung health communication campaigns that have been conducted in neighboring low-income countries [7]. A secondary objective was to examine whether ACSM awareness differed by geographic location (urban vs. rural) and gender.

## Methods

The current study utilized a cross-sectional study design to identify which components of the national ACSM campaigns were most likely to be recalled within the community. A stratified multi-stage random sampling design was used to collect data from 2,400 participants. Stage one involved randomly selecting tehsils (sub-districts) from across Pakistan, while the second stage involved randomly selecting urban union council areas and rural union council areas from each selected tehsil. The total number of localities within each selected union council was collected from the Census Organization of Pakistan. Using systematic random sampling, 43 urban and 74 rural union councils were selected from across five provinces: Punjab, Azad Jammu and Kashmir, Khyber Pakhtunkhwa, Sindh, and Balochistan. For urban union councils, each locality was further divided into nine notional parts (clusters) of equal size. Half of the selected households were randomly assigned to be 'male’ households where only males from the target cohort (18–49 years of age) were interviewed, while the other half were randomly assigned as 'female’ households where only females from the target cohort were interviewed. This approach was undertaken to ensure that an equal number of males and females were sampled, thus addressing concerns that women in Pakistani culture may be under-represented in survey-based research [8]. This process also removed the potential for interviewers to introduce selection bias into the design by arbitrarily deciding which gender to interview in each household.

Next, within each selected notional cluster, every fifth household was targeted until a total of 28 households were surveyed from that cluster. In cases where the respondent category was not available within the selected household, the adjacent household was visited. To identify the survey respondent within each targeted household, all eligible persons, regardless of gender, were listed. Then, depending on whether the household had been pre-selected as a 'female’ or 'male’ household interview, one individual from the list of household members was selected to take the survey instrument using a Kish grid table [9].

In the case of rural union councils, the area encapsulated within a union council was divided into nine notional parts of equal size and two parts were selected using a systematic sampling method. Each of these notional parts were then further divided into clusters of 150 households after making a transactional walk and having discussions with the relevant councilor/area head/village head. One cluster was then selected at random. The exception to this process was when a village formed a notional part of a union council. In this situation, the village was not broken down into smaller clusters. Within the chosen cluster, every ninth household was selected until a total of 16 households had been surveyed. The sampling methodology was then the same as that used for urban union councils.

Administration of the survey instrument was undertaken by a total of 112 enumerators under the guidance of 16 supervisors. All fieldworkers underwent extensive training, which included a background briefing on the project and its objectives as well as information about the range of ACSM activities that had been undertaken during the preceding programing period. Time was also allocated to 'workshop’ the survey instrument in order to identify potentially ambiguous wording and to ensure that all item skips were clearly understood and that the fieldworkers understood the protocols for employing and recording item probes. All instructions were recorded in an easy to follow fieldworker guide. Finally, mock interviews and in-field pilot testing were undertaken prior to the actual fieldwork.

The survey instrument assessed: (i) sociodemographic background and household information; (ii) recall of the ACSM mass-media and community-based TB campaigns; and (iii) TB knowledge and attitudes. TB knowledge and attitude were examined using three-point Likert scales, whereby 1 = disagree, 2 = neither agree/disagree, and 3 = agree. In line with Behling and Law, [10] the final version of the questionnaire was translated into the local language and then back translated to ensure equivalence of concepts and scales. Approval for the implementation of the study method and instruments was obtained through the Ethics Committee of the National TB Programme within the Government of Pakistan’s Ministry of IPC National Tuberculosis Control Program (approval number: NTPEC-MC/TBACSM/30/1/13). All participants gave their informed consent prior to completing the survey instrument.

## Results

### Sample socio-demographic profile

As shown in Table 1, respondent ages ranged from 18 to 49 years (*M* = 30.53, *SD* = 8.92). The urban and rural splits were 50.6% (*N* = 1,208) and 49.4% (*N* = 1,179), respectively, and approximately one in two respondents (53%, *N* = 1,266) had a monthly family income ≤ 10,000 rupees.

**Table 1:** **Demographic profile of study respondents (*N *= 2,387)**

**Demographic profile**	***n***	**%**
Geographic location		
Urban	1208	50.6
Rural	1179	49.4
Gender		
Male	1198	50.2
Female	1189	49.8
Relationship status		
Married	1793	75.1
Unmarried	573	24.0
Widowed/divorced/separated	21	0.9
Self-reported literacy in Urdu		
Yes	1548	64.9
No	839	35.1
Monthly family income (Rupees)		
< 1000	132	5.5
1001 – 5000	487	20.4
5001 – 10,000	647	27.1
10,001 – 15,000	389	16.3
15,001 – 20,000	156	6.5
> 20,000	280	11.7

Note: Column totals may not sum to 100% because of missing data.

### ACSM campaign awareness by socio-demographic background

Respondents were categorized as being: aware of both media and community ACSM activities (Aware_Media_&_Community_); aware of ACSM media activities only (Aware_Media_); or not aware of any ACSM activities (Unaware_Media_&_Community_). Comparative analyses involving respondents who were only aware of ACSM community activities (2.7%, *n* = 64) were not feasible because of the small size of this group. Chi square tests of independence revealed that campaign awareness varied as a function of respondents’ geographic location and self-reported literacy (see Table 2). Specifically, urban respondents were more likely to be Aware_Media_&_Community_ and Aware_Media_ than rural respondents. Similar, those who reported that they were literate were more likely to be Aware_Media_&_Community_ and Aware_Media_ than illiterate respondents. However, no gender differences were reported with respect to campaign awareness.

**Table 2:** **ACSM campaign awareness by demographic profile (*N *= 2,312)**

	**ACSM campaign awareness**						
	**Aware**_**Media**_&_**Community**_		**Aware**_**Media**_		**Unware**_**Media**_&_**Community**_		***χ***^**2**^
	***n***	**%**	***N***	**%**	***n***	**%**	
Location							21.31^***^
Urban	116	**58.3**	389	**55.9**	661	46.6	
Rural	83	41.7	307	44.1	756	**53.4**	
Gender							2.79
Male	89	44.7	340	48.9	719	50.7	
Female	110	55.3	356	51.1	698	49.3	
Literacy							78.39^***^
Literate	152	**76.4**	528	**75.9**	821	57.9	
Illiterate	47	23.6	168	24.1	596	**42.1**	

^***^p < .001.

Underlined percentages indicate adjusted standardized residual ≤ −1.96; **Bolded** percentages indicate adjusted standardized residual ≥ 1.96.

### ACSM campaign awareness by TB knowledge and attitudes

Likert-type scales, such as the ones used to assess knowledge and attitudes, can be appropriately examined using ANOVA and other parametric statistics because these analyses are robust to violations of non-linearity [11]. A series of ANCOVAs were therefore used to compare knowledge and attitudes towards TB control across Aware_Media_&_Community_, Aware_Media_), and Unaware_Media_&_Community_. The covariates for these analyses were location, gender, and literacy as these variables were found to influence ASCM awareness (see Table 2). Given the number of comparisons conducted, the alpha level for statistical significance was set at the more conservative *p* < .01 to reduce the possibility of Type I error (see Table 3).

**Table 3:** ACSM campaign awareness by TB knowledge and attitudes

**Item**	**ANCOVA**	**Post-hoc**	
	***F***	***M***	***SD***
There is no cure for TB			
Location	2.00	-	-
Gender	2.93	-	-
Literacy	9.87^**^	-	-
ACSM awareness	3.15	-	-
Aware_Media_&_Community_ (*n* = 198)	-	1.22	0.60
Aware_Media_ (*n* = 622)	-	1.24	0.64
Unaware_Media_&_Community_ (*n* = 1,291)	-	1.34	0.72
Location	0.83	-	-
Gender	0.19	-	-
Literacy	2.39	-	-
ACSM awareness	3.68	-	-
Aware_Media_&_Community_ (*n* = 197)	-	2.58	0.80
Aware_Media_ (*n* = 585)	-	2.54	0.80
Unaware_Media_&_Community_ (*n* = 1,210)	-	2.44	0.83
Location	17.77^***^	-	-
Gender	10.82^**^	-	-
Literacy	0.36	-	-
ACSM awareness	11.31^***^	-	-
Aware_Media_&_Community_ (*n* = 191)	-	1.80^ab^	0.95
Aware_Media_ (*n* = 583)	-	2.12^a^	0.94
Unaware_Media_&_Community_ (*n* = 1,200)	-	2.18^b^	0.93
Location	0.05	-	-
Gender	16.60^***^	-	-
Literacy	0.03	-	-
ACSM awareness	0.59	-	-
Aware_Media_&_Community_ (*n* = 191)	-	2.82	0.58
Aware_Media_ (*n* = 613)	-	2.83	0.53
Unaware_Media_&_Community_ (*n* = 1,265)	-	2.80	0.57
Location	0.04	-	-
Gender	0.03	-	-
Literacy	1.11	-	-
ACSM awareness	5.42^**^	-	-
Aware_Media_&_Community_ (*n* = 187)	-	2.66	0.73
Aware_Media_ (*n* = 573)	-	2.64^a^	0.73
Unaware_Media_&_Community_ (*n* = 1,149)	-	2.53^a^	0.80
TB treatments and medicines are too costly			
Location	0.29	-	-
Gender	0.05	-	-
Literacy	12.16^***^	-	-
ACSM awareness	6.23^**^	-	-
Aware_Media_&_Community_ (*n* = 194)	-	1.94^a^	0.97
Aware_Media_ (*n* = 584)	-	2.02^b^	0.95
Unaware_Media_&_Community_ (*n* = 1,175)	-	2.19^ab^	0.93
Location	1.59	-	-
Gender	18.16^***^	-	-
Literacy	2.81	-	-
ACSM awareness	6.13^**^	-	-
Aware_Media_&_Community_ (*n* = 188)	-	2.14^a^	0.95
Aware_Media_ (*n* = 550)	-	1.86^a^	0.92
Unaware_Media_&_Community_ (*n* = 1,114)	-	1.98	0.94
Location	43.29^***^	-	-
Gender	0.98	-	-
Literacy	0.01	-	-
ACSM awareness	7.92^***^	-	-
Aware_Media_&_Community_ (*n* = 188)	-	2.07^ab^	0.98
Aware_Media_ (*n* = 573)	-	1.81^a^	0.95
Unaware_Media_&_Community_ (*n* = 1,211)	-	1.75^b^	0.93
Location	11.31^**^	-	-
Gender	0.14	-	-
Literacy	5.35	-	-
ACSM awareness	14.44^***^	-	-
Aware_Media_&_Community_ (*n* = 182)	-	2.35^ab^	0.93
Aware_Media_ (*n* = 559)	-	2.11^ac^	0.95
Unaware_Media_&_Community_ (*n* = 1,172)	-	1.93^bc^	0.94
Location	6.28	-	-
Gender	2.26	-	-
Literacy	3.15	-	-
ACSM awareness	9.81^***^	-	-
Aware_Media_&_Community_ (*n* = 192)	-	2.87^a^	0.48
Aware_Media_ (*n* = 609)	-	2.78^b^	0.60
Unaware_Media_&_Community_ (*n* = 1,273)	-	2.67^ab^	0.70
Location	2.22	-	-
Gender	15.76^***^	-	-
Literacy	0.04	-	-
ACSM awareness	5.90^**^	-	-
Aware_Media_&_Community_ (*n* = 193)	-	2.80^a^	0.59
Aware_Media_ (*n* = 605)	-	2.65	0.72
Unaware_Media_&_Community_ (*n* = 1,261)	-	2.60^a^	0.76

^**^p < .01; ^***^p < .001.

Note: Common superscript letters indicate significant post-hoc pairwise comparisons.

Unaware_Media_&_Community_ cases tended to have poorer knowledge about TB and less positive attitudes towards TB treatment relative to their more ACSM-aware counterparts. Specifically, Unaware_Media_&_Community_ cases were less likely than Aware_Media_ cases to know that TB medications should be taken for 8 months to ensure a complete recovery. They were also less likely than Aware_Media_&_Community_ and Aware_Media_ cases to know that someone with a persistent cough should be referred to a TB clinic and more likely to believe that: (i) TB treatment is costly; and (ii) they do not know how to prevent or treat TB. Finally, relative to Aware_Media_&_Community_ cases, those in the Unaware_Media_&_Community_ group were: (i) less likely to know that TB cannot be transmitted by shaking hands or sharing dishes; (ii) more likely to believe that TB treatments are not readily available; and (iii) less prepared to disclose their TB status to friends or family.

Differences were also observed between the Aware_Media_&_Community_ and Aware_Media_ cases. In particular, Aware_Media_&_Community_ were more likely than Aware_Media_ cases to: (i) know that TB cannot be transmitted by shaking hands or sharing dishes; (ii) understand that TB medicines can be provided for treatment within the home; (iii) believe that TB medications are readily available; and (iv) perceive that they knew how to prevent and treat TB infections.

### Preferred sources of information about TB by socio-demographic profile

Respondents’ stated preferences for receiving information about TB were also examined to provide a guide for the programming of future ACSM activities. The most preferred sources of information about TB were broadcast media (55.0%, *n* = 1,314) and print media (16.0%, *n* = 381), followed by social referents (13.4%, *n* = 321), health workers (8.9%, *n* = 213), and community referents (6.0%, *n* = 144). There were, however, important socio-demographic differences in preferred information sources (see Table 4). Rural respondents, for example, were more likely than urban respondents to prefer receiving TB information from community referents, such as religious leaders, teachers, and local leaders. Gender differences were also apparent. Males were more likely to prefer receiving TB information from print media and community referents, whereas females preferred receiving information from social referents, such as friends and family. Finally, those who self-reported that they were literate were more likely to prefer broadcast media such as TV and radio, whereas self-reported illiterate respondents were more likely to prefer social referents.

**Table 4:** **Communication channel preferences by demographic profile (*N *= 2,373)**

**Profile**	**Communication channel**										
	**Print media**		**Broadcast media**		**Social referents**		**Community referents**		**Health workers**		***χ***^**2**^
	***n***	**%**	***n***	**%**	***n***	**%**	***n***	**%**	***n***	**%**	
Location											16.00^**^
Urban	210	55.1	675	51.4	165	51.4	54	37.5	96	45.1	
Rural	171	44.9	639	48.6	156	48.6	90	**62.5**	117	54.9	
Gender											61.34^***^
Male	221	**58.0**	671	51.1	110	34.3	95	**66.0**	91	42.7	
Female	160	42.0	643	48.9	211	**65.7**	49	34.0	122	**57.3**	
Literacy											24.36^***^
Literate	260	68.2	881	**67.0**	171	53.3	94	65.3	132	62.0	
Illiterate	121	31.8	433	33.0	150	**46.7**	50	34.7	81	38.0	

^**^p < .01; ^***^p < .001.

Note: Billboards/banners were excluded as only 14 respondents (0.6% of the sample) reported preferring this communication channel.

Underlined percentages indicate adjusted standardized residual ≤ −1.96; **Bolded** percentages indicate adjusted standardized residual ≥ 1.96.

Print media = newspapers, magazines, brochures.

Broadcast media = TV, radio.

Social referents = family, relatives, neighbors, peers.

Community referents = religious leaders, teachers, local leaders.

## Discussion

### Knowledge and attitudes related to ACSM campaign awareness

Awareness of the ACSM campaign activities was associated with improved TB knowledge and more positive attitudes about TB treatment options. Cases classified as Aware_Media_ and Aware_Media_&_Community_, for example, were more likely to recommend that someone with a persistent cough seek medical attention than those designated as Unaware_Media_&_Community_. While these findings highlight knowledge gains that may be attributable to the campaign, they also draw attention to a number of areas where deficits in TB knowledge remain. Of particular concern was the finding that Unaware_Media_&_Community_ and Aware_Media_ cases were more likely to erroneously believe that TB transmission occurs through touching personal items or sharing dishes. This belief was also identified in a smaller study published in 2006, [12] suggesting that this belief remains entrenched within the community. Misperceptions about TB transmission, particularly those that could lead to stigma and discrimination, may therefore require more targeted interventions in future ACSM activities, particularly given the potential for these misperceptions to result in delays in treatment seeking behaviors and for individuals with TB to be excluded from family and social gatherings [13].

Differences were also observed in the knowledge and attitudes of Aware_Media_&_Community_ and Aware_Media_ cases, with the former displaying greater knowledge about TB transmission vectors and treatment availability options. For example, Aware_Media_&_Community_ were more likely than Aware_Media_ to know that TB treatment was available in their community and could be provided at home by health workers or other community DOTS providers. These findings point to a potential cross-media synergy effect, with exposure to the ACSM campaign across multiple communication platforms having an additive effect with respect to TB knowledge.

The issue of cross-media synergy has attracted considerable attention within commercial marketing contexts, where the emphasis has been on evaluating the impact of cross-media effects on actual sales and determining return on investment [14]. It has, however, not received equal attention in health communication contexts. For example, although there is evidence suggesting that mass media and other community level communication activities can have an overall positive impact on targeted health behaviors, [15] the individual and additive effects of specific communication channels remain unknown. This oversight is particularly relevant given that behavior change communication campaigns are becoming increasingly multifaceted, making use of mass media, community, and interpersonal channels of communication.

Future research should therefore assess whether knowledge, attitudes, and behavioral indicators remain static or improve after exposure to campaign activities across multiple communication channels. For example, does the combination of exposure to a mass media message and participation in a community activity have greater impact on knowledge, attitudes, and behaviors than exposure that is limited to one channel alone? Given the resource limitations of implementing public health communication programs in developing countries, such information could have important implications for determining budget allocation [16] and identifying optimal returns on investment for agencies implementing ACSM activities. We would therefore argue that research is urgently needed to develop models capable of evaluating the effectiveness of cross-platform exposure of mass media, community and interpersonal communication activities. In a similar vein, public health communication campaigns may also benefit from research examining the potential for 'catalytic effects’ whereby the effects of a particular communication channel only become apparent when combined with other channels of communication [17]. The outcomes of such research have the potential to provide a major contribution to the field of TB ACSM strategic planning, implementation, and evaluation.

### Socio-demographic patterns in ACSM awareness

Another key finding of the current study was the presence of an urban–rural divide in awareness of ACSM media and community activities. Although previous studies have found poorer levels of TB knowledge, attitudes, and practices in rural Pakistan, [8,18,19] this is the first to suggest that recall of campaign messages is also lower among this cohort. This finding is particularly worrisome given the relatively large rural population in Pakistan.

ACSM campaign awareness was also lower among respondents reporting that they were illiterate. Self-reported illiterate respondents were also less likely to prefer to receive information about TB through broadcast media, suggesting that they may be ill-served by a mass media-centric campaign alone. A range of ACSM campaign activities may therefore need to be developed to ensure that all audience segments are adequately reached. This is supported by a recent study suggesting that when there are known asymmetries in exposure or use of particular media communication channels, "a non-segmented approach which utilizes one communication channel alone may unintentionally entrench pre-existing disparities in health knowledge" [20].

### Preferred media channels for TB communication

The current findings also provide key insights into how health agencies could strategically tailor their use of communication channels to reach different audience segments in Pakistan. Unlike Agboatwalla et al., [19] the current findings suggest that health workers are not a preferred source of TB information. Given the need for accurate diagnosis and ongoing monitoring of patient health, further research is needed to explore factors that may contribute to this low preference rate.

The findings also provide useful insights regarding the use of community referents in TB prevention programs within Pakistan. The findings from a recent trial intervention run in Balochistan indicated that trained religious leaders were effective in getting individuals with TB-like symptoms to visit TB clinics and resulted in a significant increase in case detection rates [21]. While this approach may have some efficacy, our findings suggest that such programs are likely to be most suited for use in rural settings and even then may have limited appeal. Indeed, only 6.0% of respondents reported preferring community referents such as religious or local leaders for receiving TB related information, with 62.5% (*n* = 90) of these living in rural locations.

### Limitations

One of the key strengths of this study was the utilization of a stratified random sampling technique that included both men and women. Nevertheless, the study was not without limitations. First, although the findings regarding the impact of the various ACSM activities were encouraging, the cross-sectional nature of the study prevented an absolute assessment of knowledge gains arising from the ACSM campaign activities. Prospective, longitudinal study designs are therefore required to more fully evaluate the potential influence of future ACSM campaign activities. A second limitation was the inability to conduct an in-depth analysis of the broad range of communication channels through which ACSM operates. Indeed, as noted previously, research is required to explore whether exposure to multiple communication channels has a synergistic or additive effect. Such effects may emerge when the campaign brand and key messages are integrated across a range of communication platforms, including broadcast media, print media, and community media and interpersonal communication activities. Research into such synergistic effects would provide valuable insights not only for TB ACSM activities but also for how best to design health communication programs in low- and middle -income countries.

## Conclusions

ACSM activities aimed at improving TB knowledge in Pakistan appear to be having some effect, with Unaware_Media_&_Community_ cases having poorer TB knowledge and less positive attitudes towards TB than their ACSM-aware counterparts. Nevertheless, awareness of TB-related ACSM activities remains suboptimal for certain segments of the Pakistan community, including rural residents and those who are illiterate. The findings also suggest that choosing between media and community communication channels need not be an either-or decision as a potential additive effect across ACSM media and community activities was identified. These findings have implications for both the development and implementation of future TB ACSM initiatives in Pakistan.

## Abbreviations

ACSM: Advocacy communication, and social mobilization; DOTS: Directly observed treatment short-course; TB: Tuberculosis.

## Competing interests

FN is the Manager of MercyCorps (Pakistan), the non-governmental organization that conducted the ACSM activities and sub-contracted the mid-term evaluation to independent advisors. FN was not involved directly with the data collection or analysis and, as such, has no competing interests. JB oversaw the qualitative component of the mid-Term evaluation but was not involved in the analysis of the quantitative data-set and, as such, has no competing interests. TT, FJN, and JDN were independent advisors on the mid-term evaluation and have no competing interests.

## Authors’ contributions

TT developed the fieldwork approach and sampling methodology, supervised the data collection and analyses, and compiled the final article. FJN and JDN conducted the data analyses, compiled the tables, and contributed to the writing of the final article. FN managed the research project, subcontracted the consultants and fieldwork teams, managed the data collection, and contributed to reviewing of the final article. JB oversaw the qualitative data collection and data analysis and contributed to reviewing of the final article. All authors read and approved the final manuscript.

## Pre-publication history

The pre-publication history for this paper can be accessed here:

http://www.biomedcentral.com/1471-2458/13/887/prepub
